# Neuromodulation of prefrontal cortex cognitive function in primates: the powerful roles of monoamines and acetylcholine

**DOI:** 10.1038/s41386-021-01100-8

**Published:** 2021-07-26

**Authors:** Roshan Cools, Amy F. T. Arnsten

**Affiliations:** 1 Department of Psychiatry, Radboud University Medical Center, Nijmegen, the Netherlands; 2 Department of Neuroscience, Yale University School of Medicine, New Haven, CT USA

**Keywords:** Excitability, Cognitive control

## Abstract

The primate prefrontal cortex (PFC) subserves our highest order cognitive operations, and yet is tremendously dependent on a precise neurochemical environment for proper functioning. Depletion of noradrenaline and dopamine, or of acetylcholine from the dorsolateral PFC (dlPFC), is as devastating as removing the cortex itself, and serotonergic influences are also critical to proper functioning of the orbital and medial PFC. Most neuromodulators have a narrow inverted U dose response, which coordinates arousal state with cognitive state, and contributes to cognitive deficits with fatigue or uncontrollable stress. Studies in monkeys have revealed the molecular signaling mechanisms that govern the generation and modulation of mental representations by the dlPFC, allowing dynamic regulation of network strength, a process that requires tight regulation to prevent toxic actions, e.g., as occurs with advanced age. Brain imaging studies in humans have observed drug and genotype influences on a range of cognitive tasks and on PFC circuit functional connectivity, e.g., showing that catecholamines stabilize representations in a baseline-dependent manner. Research in monkeys has already led to new treatments for cognitive disorders in humans, encouraging future research in this important field.

## Introduction

The primate prefrontal cortex (PFC) has the extraordinary ability to represent information in the absence of sensory stimulation, the foundation of abstract thought. The PFC generates our mental arena, and subserves our highest order functions, such as abstract reasoning, working memory, high-order decision making, planning, and organization, providing top-down control of attention, actions, and emotions. However, the PFC is remarkably sensitive to its neurochemical environment, where either depletion or overstimulation by the neuromodulators—acetylcholine, catecholamines, and serotonin—impairs its function. Neuromodulators are typically released in PFC in response to salient events, and act over a long time scale (e.g., on the order of seconds) by way of G-coupled proteins to alter the impact of nearby neurotransmission. Indeed, in the dorsolateral PFC (dlPFC), glutamate neurotransmission requires acetylcholine actions, emphasizing the importance of neuromodulation to PFC function. These powerful influences by neuromodulators coordinate our cognitive and emotional states with environmental events and our state of arousal, which can have survival value, e.g., saving energy when fatigued, or rapidly switching control of behavior to more reflexive circuits in response to danger. However, these built-in mechanisms to take the PFC “off-line” also render the PFC especially vulnerable to dysfunction. This can be particularly problematic when these signaling events are not properly regulated, e.g., due to genetic mutations and/or inflammatory insults, rendering the PFC susceptible to atrophy and degeneration. Thus, understanding neuromodulatory influences on PFC circuits can help to explain why these circuits are so often impaired in mental disorders.

Importantly, these modulatory actions appear to vary based on PFC subregion, circuit, cell-type, and receptor, with no universal actions. There are also large species differences between rodents and primates [1, 2], adding further complexity. Given these species differences, and the great expansion and differentiation of the PFC in primates [3, 4], this review will focus on neuromodulation of the human and nonhuman primate PFC by the arousal systems. The most detailed mechanistic information has focused on layer III of the dlPFC, as this layer contains the microcircuits that generate mental representations needed for working memory. As these neurons are greatly affected in schizophrenia [5, 6] and Alzheimer’s Disease [7], understanding their modulation has had particular clinical relevance, and will be a focus in this review. However, it is likely that other PFC circuits have very different molecular needs, which may relate to their specialized functions. Studies of PFC neuromodulation in humans are also reviewed, as these provided a complementary, systems-wide view, with immediate relevance to our understanding of how cognitive impairments can arise when there are disruptions in neuromodulatory mechanisms.

Please note that there are large arenas where little or no research has been performed in this field, and these discrepancies are reflected in the degree of discussion in this review. For example, there has been little research on the effects of serotonin at the cellular/molecular level in primate dlPFC, and thus this section is necessarily brief. Similarly, selective dopamine (DA) D1 receptor (D1R) agonists have been under development but have yet to be approved for widespread human use, and thus the section on neuromodulation of PFC functions in humans reflects the more frequent studies using D2R or mixed DA receptor agonists. The reader is also cautioned that methylphenidate, which is often referred to as a DAergic compound, actually increases NA as well as DA availability in the PFC [8, 9]. Finally, the reader is reminded that PFC functions are carried out as part of larger networks, e.g., involving connections with posterior cortex and subcortical structures, including striatal-cortical circuits. A review of these connections is beyond the scope of the current article, but can be found elsewhere in this special volume (e.g., Haber and Bullmore). By analogy, the putative differential consequences of neuromodulation across different brain regions are also beyond the scope of this review. Clearly neuromodulatory actions in these other nodes also contribute to overall network activity and cognitive and behavioral change.

## A brief review of PFC topography

The large, highly differentiated primate PFC is topographically organized along multiple dimensions, with more newly evolved areas situated more rostrally, and more primitive areas and functions situated more caudally [3]. Rostral and dlPFC subregions expand from marmoset to rhesus macaque to human [10, 11] suggesting that some aspects of dlPFC functions may be better studied in macaques than in New World monkeys. Imaging studies in humans have shown that more rostral PFC areas are activated by more abstract representations and manipulations (e.g., metacognition), while more caudal PFC areas are activated by simpler representations [12, 13]. Although it is challenging to study metacognition in macaques, data show that the frontal pole is also important for self-evaluation in monkeys [14]. Anatomists have long recognized that inputs to the PFC are also organized along a dorsolateral to ventromedial spectrum, with the dorsal and lateral PFC areas receiving projections from the visual and auditory association cortices, thus representing the external world, while the ventral and medial PFC areas receive inputs about stimuli impinging on or within our bodies (taste, smell, somatosensation including pain and viscera) and represent our internal world [15, 16]. For example, circuits in the dlPFC subserve visuospatial working memory, and are able to maintain a representation of visual space without sensory stimulation for many seconds. These dlPFC neurons can maintain representations even in the face of distractors, a property that may be unique to PFC [17], with projections back to the association cortices, thalamus, and basal ganglia to mediate top-down regulation of attention [18]. The dlPFC can also influence motor responding through projections back to the frontal eye fields, premotor areas and basal ganglia, including the ability to inhibit inappropriate responses, e.g., [19]. In contrast, neurons in the ventral PFC (often called orbital PFC) generate flexible representations of reward values to guide decision making, e.g., the changing reward value of chocolate or other palatable foods as one eats to satiation [20, 21]. These areas can mediate our emotional responses via connections to the medial PFC, specifically the anterior cingulate cortex (BA24) and subgenual cingulate cortex (BA25), which have outputs to hypothalamus, ventral striatum, amygdala, and brainstem to regulate our emotional responses, including autonomic regulation of the viscera [16]. The rostral areas of PFC and the dlPFC can also contribute to “top-down” emotional regulation via the ventromedial PFC (vmPFC), e.g., dlPFC area 46 connects to BA10m and BA32, and BA32 in turn projects to BA24 and BA25 to regulate emotional response [22–24].

It is also important to note that neural activity is distributed throughout cortical networks, e.g., reflected in neuronal firing and BOLD responses throughout cortex. However, lesion studies in monkeys have been particularly helpful in defining the essential and distinctive contributions of nodes in a network, e.g., the dlPFC is required for the generation of persistent firing needed for working memory and selective attention, which is then reflected in more posterior cortical areas [17, 25]; Panichello, 2021 #8302}. Thus, neurochemical actions in a single node may have ramifications for activity patterns throughout the brain.

We will see below that different PFC subregions have differing neurochemical dependencies. For example, depletion of serotonin greatly impairs the affective regulation by the orbital PFC, but has little effect on the visuospatial working memory functions of the dlPFC [26–29]. However, in-depth studies of neuromodulatory actions at the cellular level are rare in primates, and thus the reader is cautioned that there is still much to be learned in this important scientific arena.

## The arousal systems—the activity patterns of monoamine and cholinergic neurons

**Fig. 1:**
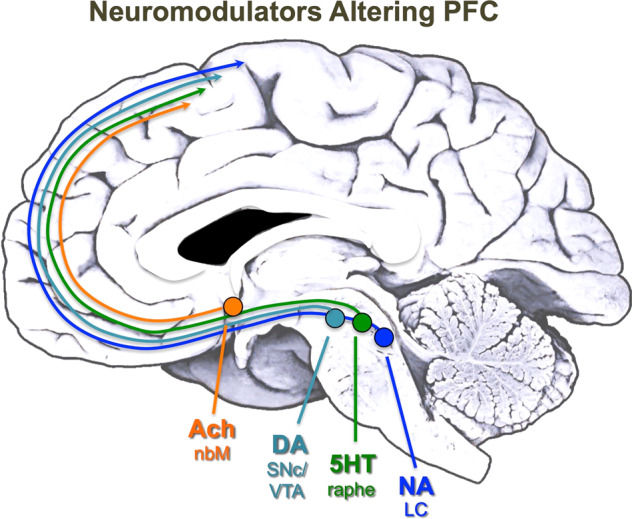
The source of monoamine and cholinergic projections to the primate PFC. The approximate position of the monoamine and cholinergic cell bodies that project to PFC are shown on a midsagittal image of a human brain. Note that many are localized more laterally, and all project to other cortical areas as well. Ach acetylcholine, nbM nucleus basalis of Meynert, DA DA, SNc/VTA substantia nigra pars compacta and ventral tegmental area, 5HT serotonin, raphe dorsal raphe, NA NA, LC locus coeruleus.

The neurons that synthesize monoamines or acetylcholine reside in the brainstem and basal forebrain (Fig. 1; see list below), with axonal projections throughout much of brain.

**Table 1:** 

Neuromodulator	Name and locations of cell bodies
NA (NA)	Locus coeruleus (LC) in brainstem [30]
DA (DA)	Substantia nigra pars compacta (SNc), ventral tegmental area (VTA), dorsal raphe [31, 32]
Serotonin (5HT)	Dorsal and median raphe [33]
Acetylcholine (Ach)	Nucleus basalis of Meynert (nbM) in basal forebrain [34]

Although previous research suggested that many of these neurons projected throughout the brain and cortex, more recent research from rodents suggests there may be much greater heterogeneity than previously suspected, e.g., with a subgroup of locus coeruleus neurons selectively targeting the PFC and not the motor cortex [35]. These monoamine and cholinergic nuclei are part of the arousal systems that control sleep vs. waking states [36]. However, most pertinent to the current review, these modulators also orchestrate brain activity within the waking state, coordinating and fine-tuning brain activity in response to external and internal events.

There are regional and laminar differences in the density and pattern of innervation in the primate PFC [37–39]. For example, DA innervation of the vmPFC is denser than in dlPFC [40, 41]. However, lower levels of innervation do not signify that an input is less important: the catecholamine innervation of dlPFC is quite modest, but selective lesioning of these axons is as devastating as removing the cortex itself [26].

PFC subregions also project back down to the cell bodies of the arousal systems to regulate brain state. The vmPFC in macaque (BA32 and BA25) projects to the dorsal raphe (5HT and DA), SNc, VTA (DA), and the LC (NE) [42] (note: only BA25 projects to the LC [42]). The orbital PFC projects to the cholinergic neurons in the basal forebrain [43], while the dlPFC projects to the LC, raphe, and midbrain DA neurons [30, 44]. Neuromodulatory neurons also receive inputs from subcortical structures, which may provide more primitive regulation. For example, the cholinergic neurons in the basal forebrain not only receive inputs form the orbital, entorhinal, and insular cortices, but also from the nucleus accumbens and hypothalamus [43]. Similarly, the NA neurons in the locus coeruleus not only receive inputs from PFC [30, 42], but from the amygdala [45], which in rodents activates the LC during stress [46]. Thus, the PFC is positioned to regulate its own neuromodulatory inputs, with the dlPFC projecting to the monoamine nuclei, and the orbital PFC projecting to the cholinergic neurons in the basal forebrain.

In general, all of these neuromodulatory arousal systems have increased firing during waking and reduced firing during deep sleep. However, there are differences related to stage of sleep, e.g., where noradrenergic LC neurons shut off completely during rapid eye movement (REM) sleep, while cholinergic neurons are activated during REM sleep (see [36] for review). Within the waking state, modulatory neurons that project to the PFC generally show increased firing to salient events. This has been demonstrated for noradrenergic neurons in the primate LC and Ach neurons in the basal forebrain, which fire to salient events, whether they are of positive or negative valence, with differing modes of baseline firing based on levels of arousal (e.g., calm vs. agitated) [47–54]. These alterations in firing, and coincident levels of release, are thought to optimize attention, e.g., by orienting attention to a novel stimulus [55], and by coordinating brain state for appropriate control of visceral and motor responses. The LC also shows enhanced firing to energize behavior for an effortful task [56]. As described below, the degree of LC firing and thus of NA release would differentially engage adrenergic receptor subtypes based on their affinity for NA, where high affinity (α2A) receptors would be engaged with moderate levels of release, and low affinity receptors (α1A, β1) with high levels of release, enhancing vs. impairing PFC function. It is possible that other modulatory systems act in this way as well. Recordings from the dorsal raphe in monkeys find phasic increases in firing to rewarding stimuli, and prolonged responses to aversive stimuli [57]. Although little is known about how serotonin alters PFC operations at its many receptors, there may be parallel actions at high affinity (e.g., 5-HT1A) vs. lower affinity (e.g., 5-HT2) receptors [58] in cortex based on the degree and pattern of raphe neuronal firing.

Recordings from DA neurons in the primate have shown discreet subpopulations with differing responses to rewarding vs. aversive events: there are “Value” neurons that increase their firing to events associated with reward, and decrease their firing to loss of reward [59]. However, there are also “Salience” DA neurons that increase their firing to both rewarding and aversive events [60, 61]. The DA neurons projecting to dlPFC appear to be “Salience” DA neurons, as DA levels increase rather than decrease in response to loss of reward [62]. It is not known if the DA neurons that project to medial PFC in primates also fire to salient events as has been shown for DA projections to medial PFC in rodent [63]. It is also possible that there is a mixture of DA “Salience” and DA “Value” neurons projecting to the primate PFC, with differing proportions in different subregions. Better understanding of this issue would require biochemical surveys of the PFC in response to gain and loss of rewards. However, fMRI studies in macaque indicate that activity in the dorsal raphe reflects changes in the global reward state [64], consistent with serotonergic neurons in mice responding to salient events [65].

## Effects of monoamine or cholinergic depletion on dlPFC vs. orbital PFC function

Neurotoxic lesions of these neuromodulatory systems have large effects on PFC functioning, although there can be qualitatively different effects based on neuromodulator and PFC subregion [66]. Extensive depletion of the catecholamines, or of Ach, but not of serotonin, markedly impairs the working memory and attention functions of the dlPFC in rhesus monkeys and marmosets [26, 27, 67, 68], including impairment of attentional set-shifting and increased distractibility [67]. We will see below that Ach plays a key permissive role in NMDAR neurotransmission in dlPFC, and that catecholamines have a powerful effect on synaptic connectivity, consistent with these depletion data. In contrast to the catecholamines and acetylcholine, serotonin depletion from the dlPFC in marmosets has little effect on performance of an attentional set-shifting task [27]. However, serotonin depletion from the ventral PFC has a very large effect [29], e.g., impairing reversal learning by failing to inhibit responding to the previously rewarded stimulus [69]. DA depletion also altered ventral PFC function, but with a qualitatively different profile. Thus, marmosets with serotonin depletion of the ventral PFC displayed stimulus-bound responding, consistent with a role in preventing competing, task-irrelevant, salient stimuli from biasing responding [28]. In contrast, monkeys with DA depletion displayed a pattern of deficits consistent with basic deficits in the associative processing of reward [28]. Reversal learning was also impaired by lesioning of the cholinergic nbM in marmosets [70, 71], suggesting that both monoamines and acetylcholine influence ventral PFC function. Overall, these data suggest that the ventral and dlPFC share a reliance on cholinergic and catecholamine actions, with the exception that the ventral PFC appears more dependent on serotonin. However, it is likely that more refined serotonergic manipulations, e.g., selective blockade of 5HT2A receptor actions [72], may reveal serotonergic contributions to dlPFC function that are not evident with global depletion. Most importantly, these studies have shown that depletion of neuromodulators can be as devastating as removing the PFC itself [26, 68], emphasizing the tremendous reliance of PFC circuits on their neuromodulatory environment.

## In-depth analyses of neuromodulation of rhesus monkey dlPFC

**Fig. 2:**
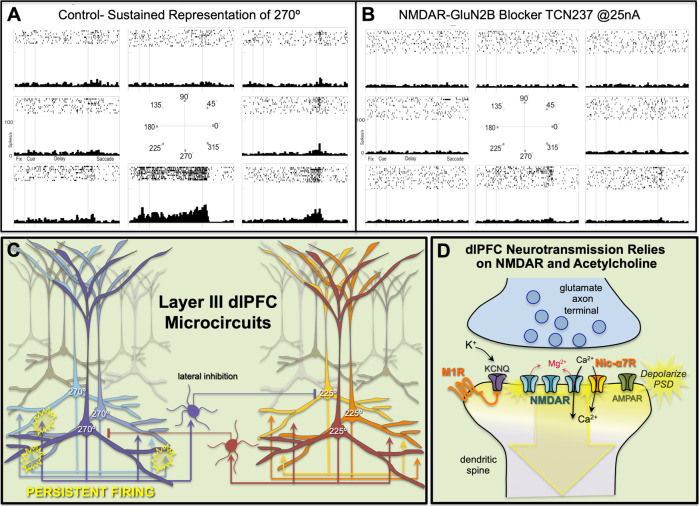
The cellular basis of mental representation by the primate dlPFC. **A** The firing patterns of a Delay cell in the rhesus monkey dlPFC during the oculomotor spatial delay response task, representing the 270° location in visual space. The neuron exhibits persistent firing across the delay epoch to the memory of a cue that had been presented at 270°, but not for other locations. **B** Iontophoresis of a drug that blocks NMDAR- GluN2B erodes the neuron’s ability to generate and sustain a mental representation of space. **C** A working model of the microcircuitry in deep layer III of dlPFC, with extensive recurrent excitation to generate persistent firing, and lateral inhibition from PV-containing interneurons to refine the contents of working memory. **D** A summary diagram of what is currently known about neurotransmission in deep layer III dlPFC recurrent excitatory synapses on spines, relying heavily on NMDAR, including those with GluN2B subunits, and cholinergic stimulation of nicotinic α7 receptors (Nic- α7R) to permit NMDAR actions by relieving the magnesium (Mg^2+^) block in the NMDAR pore. Muscarinic M1R also appear to contribute, e.g., by closing KCNQ potassium channels to depolarize the postsynaptic density (PSD). Both NMDAR- GluN2B and Nic-α7R flux high amounts of calcium (Ca^2+^), which may also help to depolarize the synaptic membrane.

The primate dlPFC is essential for working memory, and contains “Delay cells” that maintain mental representations without sensory stimulation. This fundamental operation contributes to many dlPFC functions including working memory [73], cognitive control [74], and attention regulation [17, 75]. Delay cells are able to generate and maintain persistent firing for many seconds (e.g., >15 s in Fuster’s original studies [76, 77]), representing a visual feature, a rule, or as shown in Fig. 2A, a position in visual space [73]. Strong working memory requires both persistent firing and spatial tuning such that specific information can be held in working memory stores. The cellular basis underlying Delay cell firing was discovered by Goldman-Rakic et al. in vivo [78] and confirmed by Gonzalez-Burgos et al. in vitro [79, 80]. They found microcircuits in deep layer III with extensive horizontal connections, where recurrent excitation between pyramidal cells with shared characteristic generate the persistent neuronal firing needed to maintain mental representations in working memory (Fig. 2C). They also found that these microcircuits contain fast-spiking, parvalbumin-containing GABAergic interneurons that are activated by lateral inhibition to refine the contents of working memory (Fig. 2C). The roles of additional types of interneurons in these microcircuits are still under investigation, e.g., where calretinin-containing interneurons are enriched in dlPFC and may inhibit other interneurons to boost pyramidal cell persistent firing [81]. It should also be noted that the persistent firing during a working memory task is reflected in local field potentials as increased gamma band, and reduced beta band, oscillations [82]. As local field potentials capture neuronal activity over a wide area of cortex, they are able to detect the movement of information throughout a network when the task becomes complicated by additional challenges [83].

As described in the next sections, Delay cell firing arising from recurrent excitation in deep layer III dlPFC depends on both unusual neurotransmission and neuromodulation, where the arousal systems play a critical role in permitting and shaping network firing.

### Unique neurotransmission in dlPFC

Neurons in classic circuits, such as primate primary visual cortex (V1), rely on AMPAR neurotransmission, including AMPAR permissive actions for NMDAR neurotransmission. In contrast, dlPFC Delay cells have surprisingly little reliance on AMPAR, and instead depend on NMDAR with permissive actions from acetylcholine (Fig. 2D).

In primate V1, blockade of AMPAR markedly reduces stimulus-evoked neuronal firing [84], and AMPAR are needed to process feedforward sensory input from thalamus [85]. NMDARs contribute less to processing of visual stimuli [84], although they contribute to top-down feedback regulation and local recurrence [85]. In classic circuits, AMPAR play an essential permissive role for NMDAR actions, depolarizing the postsynaptic membrane to eject the Mg^2+^ block from the NMDAR ion channel pore [86]. The rapid kinetics of AMPARs are appropriate for these circuits, where timing onset and offset are important for encoding a sensory event [87]. NMDAR with GluN2B subunits, which close slowly and flux high levels of calcium, are often extrasynaptic in classic circuits, where they contribute to excitotoxicity [88, 89].

In contrast to classic circuits, Delay cell circuits in the dlPFC have a very different function than neurons in V1: they must generate and sustain mental representations without sensory stimulation. This operation relies heavily on recurrent excitatory circuits mediated by NMDAR [90] (Fig. 2C, D), a finding predicted by computational models [91]. Thus, even low-dose blockade of NMDAR, including antagonists that selectively block those with NMDAR- GluN2A or NMDAR- GluN2B subunits, markedly reduces Delay cell firing (Fig. 2B; [91]). NMDAR- GluN2B are found exclusively within the postsynaptic density (PSD) in layer III dlPFC, consistent with their direct mediation of neurotransmission [91]. The large calcium flux by GluN2B subunits may be a key aspect of why they are needed to support persistent firing in computational models [91] and neurons [90].

In contrast to their great reliance on NMDAR, Delay cells show surprisingly subtle changes when AMPAR are blocked (Fig. 2D; [90]). This finding was unexpected, given that in classic circuits, AMPAR are essential to depolarize the PSD membrane and relieve the Mg^2+^ block within the NMDAR pore. What mechanism supplied this vital function for Delay cells in dlPFC?

### The critical role of acetylcholine for NMDAR neurotransmission in dlPFC

**Fig. 3:**
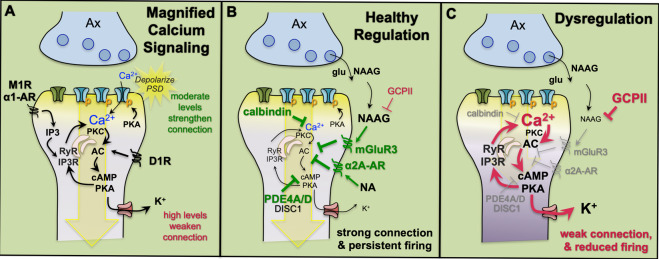
Neuromodulatory mechanisms in layer III dlPFC spines involve magnified calcium signaling that must be tightly regulated for healthy connectivity. **A** There are multiple mechanisms to magnify calcium (Ca^2+^) signaling in layer III dlPFC spines. In addition to calcium entry through NMDAR, cAMP-PKA signaling magnifies internal calcium release from the smooth endoplasmic reticulum (known as the spine apparatus in the spines, pink) through ryanodine receptors (RyR) and IP3 receptors (IP3R), which serve as internal calcium channels. Calcium in turn increases cAMP production, propelling feedforward signaling. Moderate levels of calcium strengthen synaptic connectivity via depolarization of the postsynaptic density (PSD), but high levels of calcium-cAMP-PKA signaling open nearby potassium (K^+^) channels to weaken connectivity. **B** Calcium and cAMP-PKA signaling are regulated by multiple factors, including calbindin to bind cytosolic calcium, mGluR3, and α2A-AR to inhibit cAMP production, and phosphodiesterases PDE4A and PDE4D to catabolize cAMP once it is formed. The PDE4s are anchored in place by DISC1 (Disrupted In SChizophrenia). mGluR3 are stimulated by both glutamate and NAAG (N-Acetylaspartylglutamic acid (N-acetylaspartylglutamate), which is co-released with glutamate but selective for mGluR3. **C** Loss of regulation, e.g., through genetic alterations, aging, and/or inflammation causes weakening of PFC connectivity and neuronal firing. For example, GCPII (Glutamate carboxypeptidase II) catabolizes NAAG and is increased by inflammation, reducing mGluR3 regulation and weakening synaptic connectivity. PDE4s and calbindin are lost with ageing. See text for greater details.

In dlPFC, the key permissive role for NMDAR neurotransmission appears to be played by acetylcholine acting at Nic-α7R and muscarinic M1R within the glutamate synapse [92, 93], which may depolarize the PSD to support persistent firing (Fig. 2D). Nic-α7R are ion channels that can directly depolarize the postsynaptic membrane, and can be found in glutamatergic synapses in primate dlPFC [92]. M1R also contribute and may depolarize the postsynaptic membrane indirectly [93], e.g., by closing neighboring KCNQ K^+^ channels that are also found within the glutamatergic PSD (schematized in Fig. 2B), and/or by increasing intracellular Ca^2+^ release near the synapse via IP3 signaling (Fig. 3A). These data are consistent with behavioral data showing that Ach depletion from dlPFC is as deleterious as removing the cortex itself [68]. As acetylcholine is released during wakefulness but not during deep sleep [36], these mechanisms also help to coordinate cognitive state with arousal state, permitting conscious experience during wakefulness, but may render us unconscious during deep sleep when there is no acetylcholine release.

It should be noted that high levels of muscarinic M1R signaling reduce Delay cell firing, inducing an inverted U dose/response [93–96]. This may involve M1R excitation of GABAergic interneurons, and/or excessive IP3-mediated calcium-cAMP signaling opening of K^+^ channels (Fig. 3A). These inverted U dose-response curves have also seen at the behavioral level, where systemic administration of selective M1R agonists or PAMs improve working memory at low doses, but lose efficacy and/or impair cognition at higher doses [93]. These inverted U dose-response curves can make therapeutic translation challenging, and yet are the rule, rather than exception, in PFC, including for catecholamine neuromodulation, as described in the next section.

### Unique neuromodulation in dlPFC

In classic circuits, calcium-cAMP-PKA signaling strengthens circuits, e.g., by enhancing transmitter release and/or promoting longer-term plasticity, while for dlPFC Delay cells, high levels of cAMP signaling weaken connectivity by opening potassium (K^+^) channels on spines (Fig. 3). These differences are emphasized by comparing cAMP actions in primate V1 to dlPFC. In primate V1, the cAMP-related protein, PDE4A, is localized in glutamatergic presynaptic terminals surrounding synaptic vesicle, and increasing cAMP-PKA signaling produces a linear increase in neuronal firing to visual stimuli [84]. This contrasts with the dlPFC, where cAMP-PKA signaling proteins are concentrated post-synaptically in spines. As shown in Fig. 3A, there is extensive molecular machinery for cAMP-PKA signaling to magnify calcium actions in spines. cAMP-PKA phosphorylation of NMDAR is needed to maintain NMDAR in the PSD and enhances calcium flux through NMDAR [97–99]. In addition, immunoEM has revealed a large constellation of cAMP signaling proteins on the calcium storing smooth endoplasmic reticulum (SER), known as the spine apparatus where it elaborates inside of spines [100]. cAMP-PKA signaling can increase calcium release through ryanodine receptors (RyR) and IP3 receptors (IP3R) on the SER. Calcium release into the cytosol can in turn activate adenylyl cyclase to generate cAMP, producing feedforward signaling [100]. Moderate levels of calcium-cAMP signaling are needed for Delay cell firing, likely by helping to maintain a depolarized PSD to support persistent firing (Fig. 3A). However, these spines also contain K^+^ channels (HCN, KCNQ) whose open state is increased by cAMP or PKA, needed for negative feedback and to allow dynamic shaping of network connectivity [101]. Importantly, high levels of cAMP-K^+^ channel opening markedly decrease Delay cell firing (Fig. 3A). These signaling events contribute to the narrow, inverted U dose response in Delay cell firing, as described below for both DA and NE.

Given the power of feedforward signaling events to reduce network connectivity, it is particularly important that calcium-cAMP signaling is tightly regulated (Fig. 3B). The regulation of cAMP production plays a powerful role in strengthening dlPFC network connectivity, persistent firing and working memory function. For example, metabotropic glutamate receptors mGluR3, which are primarily presynaptic in rodent, have a large postsynaptic role in primate layer III dlPFC, where they inhibit cAMP-K^+^ signaling and enhance working memory-related firing [102]. mGluR3 are stimulated not only by glutamate, but by NAAG, which is co-released with glutamate and is selective for mGluR3. NAAG is catabolized by GCPII, which is increased by inflammation [103]. These signaling mechanisms appear to have direct relevance to human cognition, as genetic insults to mGluR3 (*GRM3*) are a risk factor for schizophrenia [104], and a genetic alteration that increases GCPII production and lowers mGluR3 signaling is associated with inefficient dlPFC activity and lower IQ [105, 106]. Thus, the evolutionary expansion of postsynaptic mGluR3 signaling in PFC appears to be particularly important to human intelligence. A similar mechanism is described below, where NA stimulation of α2A-AR on spines inhibits cAMP-K^+^ channel signaling and enhances Delay cell firing and PFC cognitive functions.

Feedforward calcium-cAMP-K^+^ channels signaling is also regulated by the PDE4s that catabolize cAMP [107–109], and by calbindin, which binds calcium in the cytosol [110]. PDE4 inhibition reduces Delay cell firing [107, 109], very different from the enhancing effects on plasticity in hippocampus [111]. PDE4s are anchored to the spine apparatus by DISC1 (Disrupted In SChizophrenia) [108], which is weakened with inflammation [112, 113], or by genetic insults in a large British family with high rates of mental illness [114]. As summarized in Fig. 3C, loss of regulation by inflammation, advancing age, and/or genetic insults weakens connectivity, reduces neuronal firing, and impairs cognitive functions dependent upon the dlPFC. For example, there is evidence of reduced PDE4, calbindin, mGluR3, and α2A-AR expression with advancing age in the rhesus monkey and/or human dlPFC [109, 110, 115, 116] and a loss of Delay cell firing in aged monkeys induced by excessive calcium-cAMP-K^+^ channel signaling [117]. Dysregulated calcium-cAMP signaling is also associated with increased phosphorylation of tau [109, 110, 118]. Thus, regulation of these signaling events is key to cognitive health.

The benefits of magnified calcium-cAMP signaling near the NMDAR synapse, vs. the increased opening of nearby K^+^ channels, help to explain the inverted U dose-response curve between arousal modulators such as DA and NA and PFC cognitive functioning, as described in the following sections.

### Dopamine neuromodulation in dlPFC

The first evidence that DA was essential to dlPFC working memory function was surprisingly early, when in 1979 Patricia Goldman (not yet Goldman-Rakic) and colleagues published their landmark study showing that depletion of DA from the primate dlPFC produced as profound a deficit as removing the cortex itself [26]. Although the effective lesion depleted both DA and NE, and we now know that both are critical to dlPFC function, Goldman-Rakic’s later work showed that D1R antagonist infusions into the dlPFC also impaired working memory [119], supporting her earlier hypothesis, and opening an entire arena of research.

**Fig. 4:**
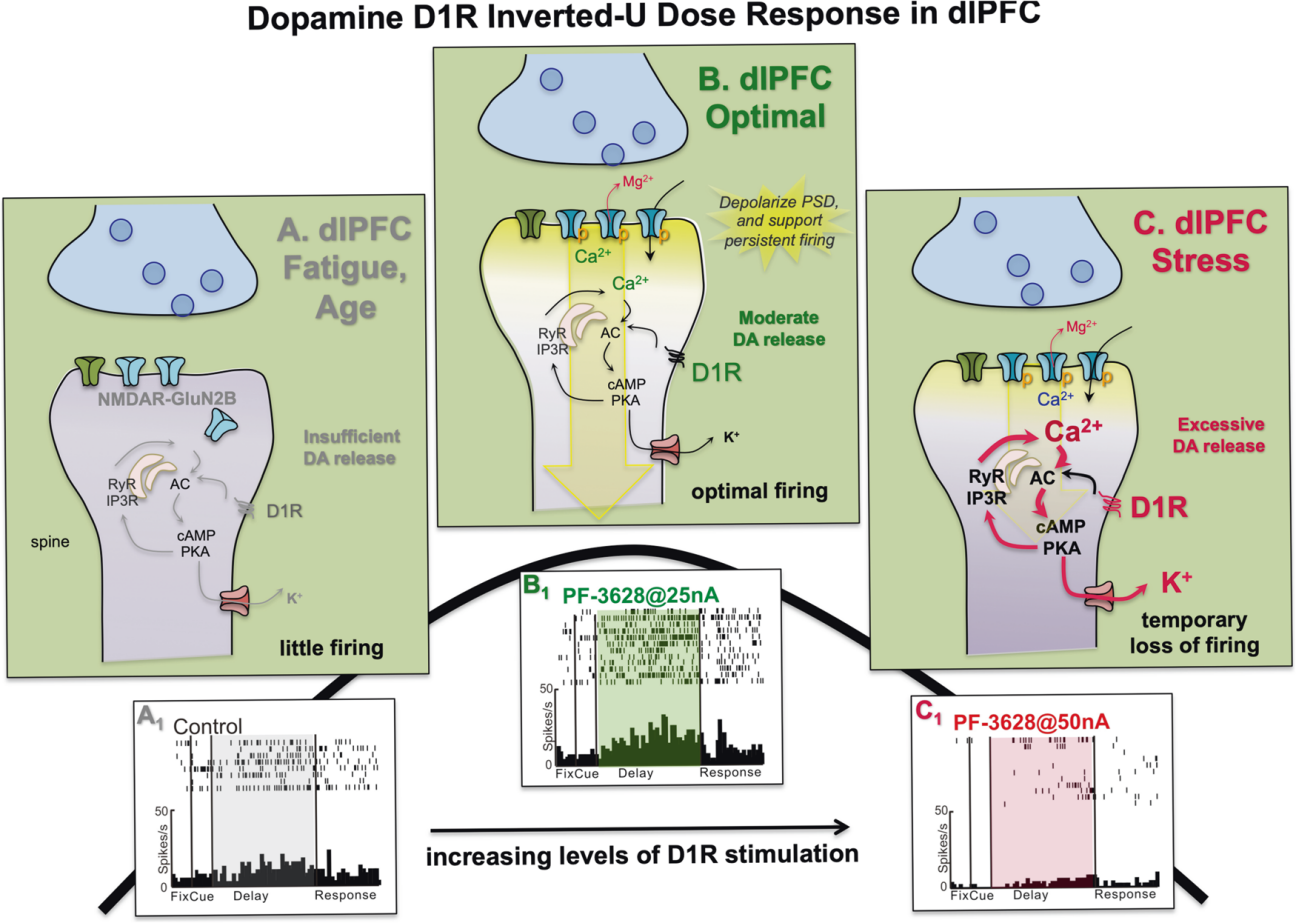
DA actions at D1R have an inverted U dose response on dlPFC Delay cell firing. **A** With insufficient DA stimulation of D1R, there is inadequate Delay cell firing (**A**_**1**_). It is hypothesized that D1R-cAMP-PKA actions are needed to phosphorylate NMDAR and maintain them within the synapse. **B** Under optimal conditions, moderate levels of D1R-cAMP-PKA activity phosphorylate NMDAR to maintain them in the postsynaptic density (PSD) and promote calcium entry through NMDAR. Moderate levels of internal calcium release would also support neurotransmission, and enhance delay-related firing (**B**_**1**_). **C** High levels of D1R-cAMP-PKA actions, e.g., as occurs during uncontrollable stress, cause a loss of Delay cell firing (**C**_**1**_) by opening large numbers of nearby K^+^ channels. Note that more subtle increases can selectively gate out “noise,” i.e., inputs from nonpreferred directions, enhancing neuronal tuning. This can be seen in Fig. 6.

DA acts at a variety of receptors, but most research works on its actions in dlPFC have focused on D1R, which are the most prominent subtype in this cortex [120], and which generally couple through Gs to increase cAMP production. In monkey and human layer III dlPFC, D1R are predominately localized on pyramidal cell spines, with a much smaller proportion expressed by parvalbumin-containing interneurons [41, 108, 121, 122]. D1R have an inverted U dose-response relationship on working memory capabilities [123–126], and on Delay cell firing [41, 122, 127, 128], e.g., as illustrated in Fig. 4. Thus, either too little DA D1R stimulation, (e.g., as occurs with high-dose D1R blockade [127] or aging [122]), or excessive D1R stimulation (as occurs with stress [124, 129] or high doses of D1R agonist [122, 128]), reduces Delay cell firing and impairs working memory. An optimal dose of D1R agonist suppressed “noise” while maintaining neuronal firing for a neuron’s preferred direction, narrowing the contents of working memory [128]. Similar inverted U effects have been seen for D1R modulation of dynamic dlPFC encoding of rules underlying cognitive control [130, 131], suggesting that these actions may generalize to dlPFC representational capabilities independent of the specific operation. As described below, the inverted U DA D1R dose response on working memory and cognitive control can also be seen in humans [132], including the narrowing of representations [133, 134], suggesting this aspect translates across species. The “narrowing” of representations by higher levels of D1R stimulation may also contribute to the increased stability, but reduced updating, of representations seen in human subjects given methylphenidate [135] (see discussion of DA effects in humans, below).

The beneficial, excitatory effects of D1R stimulation have been challenging to capture until recently, as traditional D1R agonists have had very high affinity for the receptor, whereas DA itself has relatively low affinity [122]. Thus, high affinity agonists could mimic high levels of receptor stimulation, e.g., as occurs with stress, but could not mimic the more subtle actions that occur under nonstress conditions. The reader should be cautioned that most of the research described in this section was performed with high affinity D1R agonists that do not mimic the effects of endogenous DA, although they appear to capture the effects of high levels of endogenous DA release, e.g., as occurs with stress exposure. The recent creation of low affinity D1R agonists that better mimic DA have allowed the first, direct evidence of DA D1R excitatory actions on Delay cell firing [122], e.g., as seen in Fig. 4B. D1R have been shown to enhance NMDAR actions in rodent PFC, e.g., via phosphorylation of NMDAR to maintain them in the PSD [97, 98] and to enhance calcium flux [99]. It is likely that these actions also occur in primate layer III dlPFC, where PKA-phosphorylated NMDAR can be seen in the PSD using immunoEM [90]. These actions may contribute to the beneficial, excitatory effects of D1R stimulation (schematically illustrated in Fig. 4A, B).

With increasing levels of D1R stimulation, cAMP signaling opens K^+^ channels to gate out network inputs, preferentially reducing the neuron’s response to nonpreferred directions (“noise”), while retaining delay-related firing to preferred inputs (“signal”) [128]. This narrowing of the mental representation may be helpful in refining representations in the face of interference. However, further increases in D1R stimulation, e.g., as occurs with uncontrollable stress, reduces neuronal firing to signals as well as noise, eroding rather than refining neural representations. High levels of D1R stimulation, even with the lower affinity agonist, cause a marked loss of Delay cell firing (Fig. 4C) [122, 128] and working memory deficits with either local or systemic D1R agonist administration [41, 125, 126, 131]. High levels of D1R stimulation also render pyramidal cells more vulnerable to distractors [136]. Conversely, a D1R antagonist can reverse the effects of uncontrollable stress on working memory performance [124].

The intracellular signaling events that mediate the detrimental effects of high-dose D1R agonists on Delay cell firing and working memory are illustrated in Fig. 4C; the loss of dlPFC Delay cell firing can be blocked by a PKA inhibitor [128] or an HCN channel antagonist [137]. Thus, the ability of D1R to strengthen vs. weaken synaptic activity may be related to both the degree of cAMP-calcium signaling, and the location(s) of D1R on the spine membrane on a particular network connection on a spine, where D1R within/near the PSD may be more likely to strengthen firing, while those at more distant locations from the PSD but close to HCN and KCNQ channels on the spine may promote the gating of network inputs [41], and with higher levels (e.g., during uncontrollable stress), the loss of dlPFC network connectivity, neuronal firing, and cognitive function (Fig. 4C).

D2 receptors have more limited expression in primate dlPFC and are focused in layer V neurons [120]. Alterations in D2 receptor stimulation, using compounds that interact with both D2 and D3 receptors, have little effect on Delay cell firing, but have large effects on the firing Response feedback neurons, cells that may respond to the corollary discharge that an eye movement as occurred [138]. Stimulation of D2/D3 receptors increases the amplitude [138] and speed [41] of the response, while blockade does the converse. As corollary discharge can be a neural signal of a self-generated movement or thought, distortions in D2R signaling may alter this operation and contribute to delusions and/or hallucinations, e.g., attributing actions to an outside agent. It should be noted that if the Delay cells and Response cells were added together, as is typical of most neurophysiological studies that average all neurons in a population response, we would observe that both D1R and D2R enhance working memory-related neuronal firing, albeit with the D1R enhancement limited to modest levels of D1R stimulation, while higher levels suppress firing.

Many of DA’s influences on dlPFC neuronal firing during spatial working memory can also be seen during other cognitive operations [131, 139], e.g., where high doses of D1R stimulation are suppressive (although “signals” can be maintained and accentuated if the dose is not too high), while D2R stimulation is often excitatory, especially in regard to motor responses [130]. For example, high levels of D1R stimulation render pyramidal cells more vulnerable to distraction [136]. It remains to be seen whether new, lower affinity D1R agonists will strengthen rather than weaken resistance to distraction, as suggested by experiments that deplete endogenous DA [67]. Recordings from dlPFC neurons in monkeys representing and evaluating numbers also show that DA can enhance rule encoding, where high levels of D1R stimulation suppress general neuronal firing but increase responses to the preferred rule, while D2R stimulation excited neuronal firing in general while suppressing responses to the nonpreferred rule [140]. D2R stimulation also enhanced persistent firing of numerical representations by dlPFC neurons [141]. It should be noted that the experiments on D2R mechanisms in primate dlPFC generally utilize the agonist, quinpirole, which stimulates D2, D3, and D4 receptors [142]. As D2R and D4R are concentrated on interneurons in dlPFC ([143], and see next paragraph), some of the excitatory effects of quinpirole in these studies may arise from D2R/D4R inhibition of interneurons, thus disinhibiting pyramidal cell firing [141].

The primate frontal eye fields (FEF) are situated immediately caudal to the dlPFC, and appear to be modulated by DA in a similar manner. These studies also found that D1R predominated on pyramidal cells compared to interneurons, while D2R predominated on interneurons [144]. D1R were particularly concentrated on pyramidal cells with long-range projections back to the visual extrastriate cortices, which coordinate eye movements with visual attention [144]. Blockade of DA D1R stimulation in the FEF enhanced FEF influence on V4 processing of visual stimuli, consistent with higher levels of D1R stimulation weakening neuronal function in both dlPFC and FEF [145]. Although these data suggest that DA D1R may have similar effects across cortical areas, it is likely that there are multiple differences as well, and thus extensive research will be needed to learn how DA influences distinct circuit operations across the primate cortex. Additional research is also needed to understand the contributions of D3, D4, and D5 receptors. It is noteworthy that the D4 receptor actually has higher affinity for NA than DA [146], a reminder that our terminology does not respect the overlap in catecholamine actions.

### Noradrenaline neuromodulation in dlPFC

**Fig. 5:**
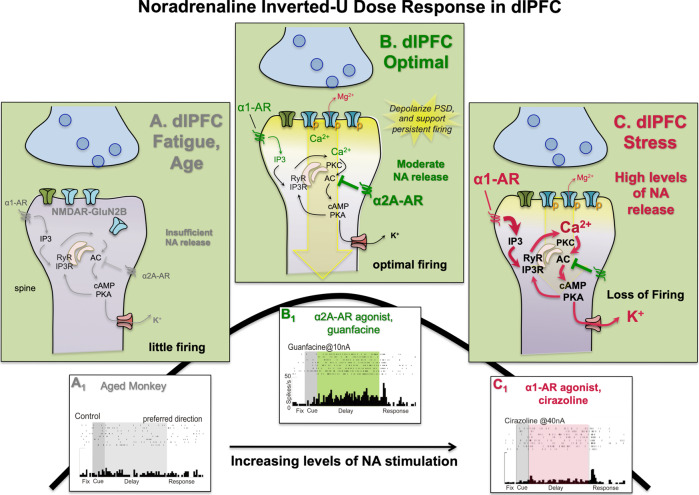
NA has an inverted U dose response on dlPFC Delay cell firing through differential actions at α2A-AR vs. α1-AR. **A** With insufficient NA stimulation of α2A-AR, there is inadequate Delay cell firing (**A**_**1**_) due to excessive cAMP-PKA-K^+^ channel signaling, which weakens connectivity. **B** Under optimal conditions, moderate levels of NA engage high affinity α2A-AR to reduce cAMP-PKA-K^+^ channel opening, strengthening connections and enhancing delay-related firing (**B**_**1**_). **C** High levels of NA release, e.g., as occurs during uncontrollable stress, engage low affinity α1-AR to increase calcium drive on cAMP-PKA-K^+^ channel opening, weakening connections and reducing delay-related firing (**C**_**1**_). Transient increases in NA release may engage α1-AR and β1-AR to disconnect circuits to allow reorganization of network configurations for changing environmental demands.

Although initial research on catecholamine actions in dlPFC focused on DA, it is now appreciated that NA has just as powerful effects on dlPFC physiology and function with a parallel, inverted U dose response (Fig. 5). Importantly, the separation of beneficial vs. detrimental actions at separate adrenergic receptor subtypes has allowed for rapid translation to human therapeutics. NA has differing affinities for its receptors, with highest affinity for α2A-AR, lower affinity for α1A-AR, and lowest affinity for β-AR [147]. Thus, the amount of NA release can determine which receptors are engaged, and whether dlPFC Delay cell firing is enhanced or weakened. As reviewed above, NA is released according to arousal state, with little or no LC firing during sleep, phasic firing to relevant stimuli during nonstress waking, and high levels of spontaneous firing during stress [36, 51, 52]. The data suggest that α2A-AR, but not α1-AR or β-AR, are predominately engaged during nonstressed waking when there are moderate levels of NA release [148], while α1A-AR become engaged with higher levels of NA release during stress exposure [149]. Although previous research has emphasized the presynaptic role of α2A-AR, it should be noted that the enhancing effects of NA in PFC occur at postsynaptic α2A-AR [107, 150, 151], which are localized on spines near glutamate synapses, positioned to strengthen connectivity [107]. As α2A-AR strengthen dlPFC network connectivity, and α1A-AR weaken connectivity and dlPFC firing, these changes in arousal state can cause large changes in cognitive state.

As illustrated in Fig. 5A, B, stimulation of α2A-AR under nonstress conditions strengthens dlPFC Delay cell firing via inhibition of cAMP-K^+^ channel signaling [107]. Conversely, blockade of α2A-AR markedly reduces Delay cell firing that can be reversed by blocking HCN channels [107, 152]. Similar effects have been seen on working memory performance with local or systemic administration of α2A-AR agonists or antagonists [148, 150, 153, 154]. The α2A-AR selective agonist, guanfacine, is able to improve working memory with the least sedative side effects [153, 155, 156], and also improves other PFC cognitive operations such as attention regulation [157], associative learning [158], delayed discounting [159], and reversal learning dependent on the ventral PFC [160]. Guanfacine is in widespread use for treating PFC disorders based on research in animals [161]. It is noteworthy that the enhancing effects of methylphenidate on working memory in monkeys involve increased α2A-AR, as well as D1R actions [9]. Thus, α2A-AR stimulation likely contributes to the increased stability of representations produced by methylphenidate in humans [135].

In contrast, stimulation of α1-AR reduces Delay cell firing and impairs working memory performance. As seen in Fig. 5C, iontophoresis of an α1-AR agonist into the dlPFC reduces Delay cell firing [162, 163], and local infusion of an α1-AR agonist into dlPFC impairs working memory performance [154]. Recent data suggest that lower levels of α1-AR stimulation may sometimes enhance dlPFC neuronal firing, and that this may be related to presynaptic α1-AR positioned to enhance release, and/or to modest increases in internal calcium release in spines [163]. However, higher levels of α1-AR stimulation produce profound decreases in dlPFC Delay cell firing (Fig. 5C), and this is mediated through calcium-PKC signaling [162]. Brief pulses of α1-AR stimulation may help to weaken existing network connections to promote network reconfiguration, e.g., in response to an unexpected event [164]. However, sustained high levels of α1-AR stimulation contribute to stress-induced PFC dysfunction [162]. These findings in animals help to explain why α1-AR antagonists such as prazosin are used to treat PTSD [165].

Less is known about how β-AR stimulation alters dlPFC Delay cell firing and dlPFC function. Infusion of a general β-AR antagonist into the monkey dlPFC had no effect on working memory under nonstress conditions, suggesting that these actions do not have a large influence during normal arousal conditions [148]. However, this could be an inaccurate conclusion if β1-AR and β2-AR have opposite effects on dlPFC function. Systemic administration of a β1-AR antagonist improves PFC working memory function in monkeys, suggesting that these receptors may have detrimental actions [166]. However, further research is needed to understand the role of β-AR in dlPFC.

### Serotonin modulation of PFC

Little is known about serotonin’s cellular and molecular actions in primate dlPFC. Although researchers have found no effect of serotonin depletion in dlPFC on working memory performance [26, 27], more detailed dissection of serotonin receptor actions provides a different picture. Iontophoresis of a 5HT2A receptor agonist accentuated the spatial tuning of Delay cells by increasing firing for preferred target locations and/or reducing firing for nonpreferred locations, while an antagonist had converse actions [72]. These data emphasize that negative findings with depletion experiments do not mean that a modulator has no effect, but rather that there may be complex underlying receptor actions. Much greater research is needed in this area, especially given the lesion data indicating that serotonin is critical to ventral PFC function [27]. For example, how do different serotonin receptor subtypes alter the functioning of BA25, and are there specific receptors that can quiet BA25 activity and possibly have anti-depressant actions? This is an arena where marmoset research may be particularly helpful.

### Overall summary of nonhuman primate dlPFC modulation

**Fig. 6:**
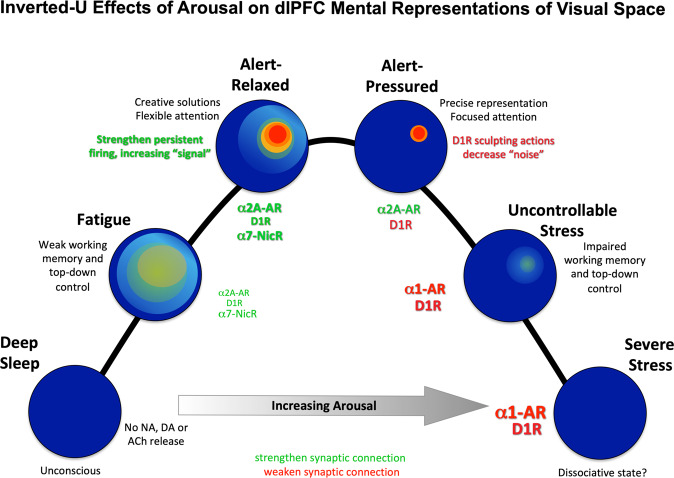
Inverted U effects of the arousal modulators on network generation of mental representations of visual space held in working memory. During deep sleep, there is no release of Ach, and little monoamine release, and thus NMDAR circuits in layer III dlPFC have no NMDAR neurotransmission, contributing to an unconscious state. In an awake but fatigued state, cholinergic release allows NMDAR transmission, but connectivity and lateral inhibition are weak, creating diffuse, weak representations. With optimal arousal in a relaxed state, there is strong connectivity and representations, e.g., from Nic-α7R and α2A-AR stimulation; and with increased D1R (e.g., due to pressure) there is also increased sculpting of “noise,” narrowing the representations held in working memory. Thus, different cognition operations may have differing optimal chemical states. With increased uncontrollable stress, high levels of D1R and α1-AR stimulation erode representations. We speculate that extreme stress may disconnect dlPFC circuits sufficiently to create a dissociative state.

Overall, research on neuromodulators shows that they have a profound influence on dlPFC function in monkeys, with an inverted U dose response that is consistent with the changes in dlPFC function with alterations in arousal state (summarized in Fig. 6). A more nuanced understanding of the inverted U has also emerged, with the understanding that what an “optimal” neurochemical state depends on the cognitive operation being performed. For example, wider mental representations (e.g., strong α2A-AR, lower D1) may benefit operations where broader network connections are useful (e.g., creative integration), whereas more narrowed, precise representations (higher D1R) may benefit tasks requiring precision and focus (e.g., math problems). We have also learned that loss of regulation of these powerful signaling events, e.g., by genetic insults or with advancing age, contributes to dlPFC deficits in cognitive disorders (Fig. 3C), emphasizing their importance to human health.

## Effects of monoaminergic and cholinergic manipulations of human PFC function

Research in humans necessarily involves global manipulations, such as systemic drug treatment, or studies of genotype, on cognitive functioning and brain activity patterns. Available pharmacological agents safe for human use often have nonselective actions, further complicating mechanistic interpretations. Nonetheless, the data indicate that neuromodulators have powerful effects on PFC cognitive functions in humans that can be consistent with that seen in monkeys. For example, muscarinic and Nic-α7R blockade impairs working memory in humans [167], as well as monkeys [93], emphasizing the importance of acetylcholine to dlPFC function. Similarly, methylphenidate, which increases both DA and NA availability in the PFC, enhances the stability of dlPFC representations, rendering them resistant to distractors [135], consistent with D1R and α2A-AR increasing dlPFC delay-related firing in monkeys described above. Indeed, the widespread use of the α2A-AR agonist, guanfacine, to treat PFC disorders such as ADHD, arose from this work in monkeys [161]. The following is a review of monoamine and cholinergic influences on human PFC cognitive functioning, where cognitive tasks can be more readily elaborated to assess a wider range of PFC functions.

### Effects of DA on human PFC function

Experimental work with human participants has substantiated the necessity of DA for working memory and associated delay-period activity in the PFC [135, 168–181], as well as other cognitive functions such as future planning and cognitive flexibility [182–185].

A key challenge for human research on PFC DA function has been the limited availability of selective DA D1 receptor agents [186]. However, an important role of for D1R stimulation in human working memory is substantiated by PET imaging with D1R-selective ligands. For example, a simultaneous PET/fMRI study with the D1R-selective PET ligand [11C] NNC112, in 29 healthy volunteers by Roffman et al. [187] has revealed that cortical D1R density predicted working memory-related decoupling of frontoparietal and default networks, which respectively manage task-related and internal stimuli. Specifically, lower cortical D1R density was associated with greater decoupling. These findings concur with the hypothesis that cortical dopamine signaling controls network dynamics to redirect cognitive resources to working memory. Moreover, the findings echo neuromodulatory effects of D1R signaling at the level of cortical microcircuits. As reviewed above, in nonhuman primates, optimal levels of D1R receptor stimulation suppress task-irrelevant inputs onto dendritic spines through a variety of mechanisms, including excitation of fast-spiking interneurons and cAMP-mediated effects on HCN channels. An intriguing target for future research is the cellular basis of network (de)coupling by D1R stimulation during working memory.

These findings also substantiated earlier PET work with the same ligand showing that an increase in dlPFC D1R is associated with working memory impairment in schizophrenia (although not healthy volunteers), which was interpreted as a compensatory upregulation in response to chronic deficits in DA tone, but could also be a sign of excessive DA D1R signaling impairing working memory early in the disease process [188, 189]. Reduced D1R availability has also been linked to cognitive deficits with advancing age [190], although this may reflect the loss of spines in dlPFC with age [191], as this is the main site of D1R localization [121].

A role for D1R stimulation in working memory was also suggested by early work with the first generation of selective D1R agonists dihydrexidine (DAR-0100) or its active enantiomer (DAR-0100A) in patients with SCZ and SCZ spectrum disorders. This work showed that it increased perfusion of PFC and enhanced performance on classic working memory tasks, like the n-back [192]. Moreover, very recent work with novel selective partial, non-catechol D1 receptor agents that avoid the major side effects associated with prior D1R agonists (e.g., on blood pressure), like PF-0641256, has shown effects on cost-benefit decision making, reversal learning, and Pavlovian control of responding depending on baseline working memory capacity [186]. For example, higher doses of PF-06412562 improved reversal learning only in individuals with low baseline working memory capacity. This development opens avenues for further unraveling the precise computational and neural mechanisms of D1R effects on working memory in healthy volunteers. The evidence for inverted U-shaped effects also in humans further emphasize the importance of taking into account his individual variability when aiming to isolate these effects.

**Fig. 7:**
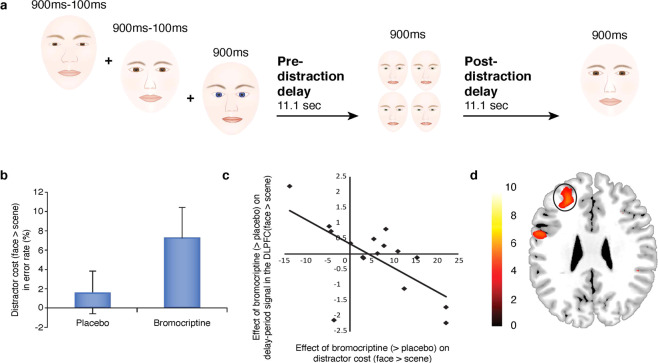
Effects of the DA D2 receptor agonist on delay activity after distraction in healthy volunteers. Adapted from Bloemendaal et al. [168] with permission. **A** Schematic diagram of the delayed match-to-sample working memory task. During the encoding phase, subjects were presented with three face stimuli. After an 11.1 s delay period, they were shown a face or scene distractor. Subjects pressed a right or left button to indicate whether this stimulus matched a pre-learned target face or scene. After another delay period of 11.1 s, the probe stimulus appeared and the subject made a left/right button press to indicate whether this probe stimulus matched one of the encoded stimuli. **B** Bromocriptine significantly increased the distractor error cost: participants made more errors when the distractor category was congruent with the dimension of the encoding stimuli (i.e., faces vs. scenes). **C** Data extracted from the dlPFC cluster that exhibits a correlation between bromocriptine-related increases in the distractor cost and bromocriptine-related decreases in delay-period activity after congruent (face) vs. incongruent (scene) distractors. **D** Locus of correlation between drug effect on behavioral distracter cost and drug effect on neural distracter cost in dlPFC. The bar indicates *T* values and figures are thresholded for a *T* value of 3.79, corresponding to a *p* value of 0.001 uncorrected for multiple comparisons.

Nevertheless, it is fair to say that the majority of the evidence for DA’s role in modulating human working memory comes from studies investigating either nonselective catecholaminergic drugs, such as methylphenidate, or D2 family agents that alter D2, D3, and often D4 receptors. Thus, while nonhuman primate research has highlighted in particular a key role for the DA D1R in dlPFC mediation of working memory, human work reinforces observations from work with rodents that DA can also modulate PFC functions, including working memory, set-shifting and reversal learning, via modulating dopamine D2R [193]. One possibility is that pharmacological stimulation or blockade of D2R alters D1R stimulation indirectly, via action at presynaptic D2R, thus eliciting changes in autoregulation of synthesis and release leading to paradoxical effects on DA tone. However, the functional consequences of D1R and D2R stimulation, at least as measured in vitro in rat mPFC, are quite different [194]. According to dual-state theory of PFC DA [195], which is grounded in computational modeling of biophysical effects of DA in rodent medial PFC, the D1R mode, associated with intermediate levels of DA, is accompanied by distractor-resistant stabilization of working memory representations, while the D2R mode, associated with either very low or very high DA levels, is accompanied by flexible updating of working memory representations. This hypothesis is supported by pharmacological neuroimaging work with human volunteers, showing that D2R stimulation, with the D2R agonists bromocriptine or cabergoline, indeed reduces the distractor-resistance of working memory representations by disrupting delay activity in dlPFC, selectively after distraction (Fig. 7) [168]. This impairment may reflect either stimulation of postsynaptic D2 receptors, shifting the PFC into a flexible D2R mode, and/or, as described above, reduced DA release and D1R stimulation in dlPFC due to bromocriptine actions at autoreceptors.

As is the case for D1R effects in nonhuman primates, so do effects of DA D2R drugs in humans also depend on the baseline state of the agent [196–200]. For example, the effects of bromocriptine and similar agonists on PFC function interact with the subject’s baseline working memory ability with the drug improving cognition in subjects with lower baseline working memory abilities in the “un-drugged” state, while worsening cognition in those with higher baseline working memory capacity [169, 171, 173, 180, 201]. Such span-dependent drug effects are seen not just for classic dlPFC tasks, such as those measuring set-shifting, working memory updating [202, 203], and working memory retrieval, but also for tasks measuring functions more commonly associated with (ventral) striatal regions, such as reinforcement learning [184, 203] and Pavlovian biasing of instrumental responding [204]. Thus, effects of DArgic drugs can, at least partly, be predicted from the initial state of the individual, an observation with clear clinical implications for individual tailoring of DArgic drug therapy.

What is the mechanistic basis of this interaction between baseline state and DA drug effects on working memory capacity in humans? It likely relates to the endogenous levels of DA neurotransmission, e.g., where those with inadequate endogenous DA levels and weaker cognition are helped by drug, and those with optimal endogenous DA and cognition are impaired by drug treatment. This follows from findings showing that effects of DArgic drugs, like the D2 receptor agonist bromocriptine, the D2 receptor antagonist sulpiride, the catechol-O-methyltransferase (COMT) inhibitor tolcapone, and the catecholamine reuptake blocker methylphenidate, can all be predicted from baseline levels of DA, as indexed with [18F]FDOPA or [18F]FMT PET [200, 205, 206], or by genetic differences between individuals, e.g., in the Val^158^Met polymorphism in the COMT gene or the Taq1A polymorphism of the DRD2 gene [134, 207–210].

Where DA-induced improvements of spatial tuning during working memory are accompanied by suppressive effects on dlPFC activity in nonhuman primates [128], so is working memory improvement after DA-enhancing drug administration in human functional imaging studies accompanied by reductions in dlPFC activity [133, 207, 211, 212]. Moreover, where too much D1 receptor stimulation in nonhuman primates, e.g., due to stress, can elicit a quelling of PFC activity and a blocking of all new input to the PFC, thus leading to perseverative responding [123, 125, 127], so can (nonspecific) dopaminergic drug administration in human subjects with already high levels of dlPFC DA also promote perseverative responding and maladaptive, inefficient increases in BOLD signaling [207]. Findings from two small sample DA PET studies demonstrating a positive link between working memory capacity and baseline DA synthesis capacity, as measured with [18F]FMT PET imaging [213, 214] further substantiate the hypothesis that the interaction between DA drug effects on dlPFC function and working memory span reflects an inverted U-shaped relationship with individual differences in baseline levels of striatal DA synthesis capacity, although studies with larger sample sizes are required to substantiate the evidence for such a link.

The dual-state theory of prefrontal cortex by Durstewitz and Seamans (2008) might also relevant here when accounting for the interaction between baseline dopamine levels and DA drug effects, given that D1 and D2 receptors have been suggested to have differential (respectively less and more) affinity [215] with opposite functional effects [194, 195]. A more recent biophysically realistic model that takes into account a D1R gradient across the rostrocaudal hierarchy in PFC (with more D1R in more rostral PFC) captures the inverted U-shaped dependence of working memory on DA as well as the filtering of irrelevant stimuli by enhancing inhibition of pyramidal cell dendrites [216]. This filtering may also occur through D1R-cAMP-K^+^ channel gating on spines (see above), as pyramidal cells and spines are the predominant location for DA synapses and D1R in human as well as monkey PFC [121, 217]. The rostral-caudal gradient is particularly intriguing given that more rostral PFC areas have been implicated in maintaining more abstract representations [218, 219]. Thus, rostral D1R stimulation might be in a perfect position to strengthen the most abstract goals, while also allowing flexible updating at a lower level of the computational hierarchy.

A final key point is that, through top-down control, the PFC can shape the responses of DA neurons in the midbrain and DA release [220–222]. Just as rodent medial and orbitofrontal cortex shape signals of midbrain DA neurons [223, 224], so are BOLD responses in human striatum shaped by an internal model of the task environment in working memory, putatively computed at the level of cortex [225–227]. In humans, there is also direct evidence for a role of the PFC in controlling DA release. This evidence comes from work combining transcranial magnetic stimulation with [^11^C]raclopride PET, even showing topographical specificity, with DA release in specific subregions of the striatum being controlled by regions in PFC that are strongly connected with those striatal regions in a topographically selective manner [228–231].

### Effects of noradrenaline on human PFC function

The LC NA system has been proposed, like cortical DA, to contribute to working memory with moderate (phasic firing) levels of NA strengthening representations held in memory stores and high (tonic firing) levels of NA weakening them [147, 232]. This is likely due to differing degrees of NA release engaging with different subtypes of adrenergic receptors that have opposing effects on dlPFC connectivity and neuronal firing. As summarized in Fig. 5, iontophoretic recordings from monkey dlPFC indicate that lower level, phasic NA release engages high affinity α2A-AR to strengthen dlPFC recurrent excitatory connections underlying mental representations [107], while higher tonic levels of NA release would engage lower affinity α1-AR and reduce weaken connectivity and representations [163].

In concert with this proposal are data from studies with humans showing that the α2A-AR agonist, guanfacine, can improve a variety of PFC cognitive operations in both healthy human subjects and patients with PFC deficits (reviewed in [161]), while the α1-AR antagonist, prazosin, is used to treat PTSD (reviewed in [165]). Guanfacine is able to restore PFC top-down control over attention, action, and emotion [233, 234], and recent work finds that guanfacine can facilitate emergence from delirium [235], consistent with α2A-AR actions increasing PFC network connectivity [107]. Guanfacine treatment improves the ability to resist distraction in monkeys [157, 236] and humans [237], similar to methylphenidate [135], raising the possibility that the enhancing effects of methylphenidate involve increased endogenous α2A-AR stimulation, as seen in monkeys [9]. The NA reuptake blocker, atomoxetine, has also been shown to increase the functional connectivity of the dlPFC in relationship to working memory capacity [238], although this compound increases DA as well as NA levels in the PFC [239]. Recordings from monkeys show that atomoxetine has an inverted U dose response on delay cell firing, with low doses enhancing firing via enhanced α2A-AR stimulation, but slightly higher levels reducing firing via increased α1-AR stimulation [9]. These mixed effects may help to explain some of the complexities in the literature using this compound.

Moreover, these data have relevance for the adaptive gain theory, according to which different modes of NA transmission regulate the tradeoff between exploitation and exploration [240]. In this model, a phasic mode promotes exploitative behavior and focused attention by facilitating processing of task-relevant information, where top-down control may come from dlPFC inputs [30]. In contrast, increasing the tonic mode promotes behavioral disengagement and divided attention, thus allowing potentially new and more rewarding behaviors to be explored. In the model, the transition from the phasic to the tonic NA mode is controlled by specific regions in the PFC, e.g., the orbitofrontal cortex and/or the ACC, which in turn control the firing of NA neurons in the brainstem in a top-down manner.

Arnsten et al. showed that the spines of layer III dlPFC pyramidal cell networks express the molecular machinery to allow rapid changes in network connection strength (termed Dynamic Network Connectivity) by which neuromodulators such as NA can flexibly alter network configurations, e.g., based on environmental demands [100, 101, 241]. Although this work was based on physiological recordings in monkeys, recent fMRI studies in humans also show that enhanced NA signaling with atomoxetine can alter cortical network organization during the resting state [242], or during working memory [238]. This work has demonstrated that atomoxetine reorganizes the functional connectome as a function of cognitive demands, altering the balance between network-level segregation and integration [242]. Atomoxetine potentiated network segregation during rest, but, potentiated network integration during a classic n-back working memory task. Such findings have general relevance, e.g., in the context of acute stress, where stress-induced increases in NA activity have been argued to prompt large-scale neural network reconfiguration [243, 244] (and see below), consistent with the dynamic changes in synaptic strength based on α2A-AR vs. α1-AR predominant actions.

These findings are also reminiscent of earlier theoretical and modeling work, suggesting that NA acts as a “neural interrupt signal” that alerts the learner to an unpredicted change in the learning environment [245] and performs a “network reset” [164]. Noradrenergic “neural interruption” also surfaces as improved response inhibition in stop-signal tasks [246], which is accompanied by increases in connectivity of the inferior frontal cortex, reflecting in part greater sensitivity to afferent inputs [247–250]. The enhanced connectivity within the inferior PFC following atomoxetine may arise from increased NA stimulation of α2A-AR strengthening synaptic efficacy (see above), as atomoxetine enhances dlPFC neuronal firing via α2A-AR actions [9]. Consistent with this hypothesis, the α2A-AR agonist, guanfacine, improves stop-signal performance in monkeys [251], and in abstinent cocaine addicts [252], and improves impulse control in monkeys [159] and patients with ADHD [253].

The notion that increased (tonic firing) cortical NA is particularly important for explorative modes of behavior concurs with recent neural models [254] suggesting that noradrenergic increases in cognitive flexibility can be accounted for by changes in the uncertainty of an internal model of the environment [240, 245]. This account is grounded in the observation that environments characterized by high volatility benefit from higher levels of perceived uncertainty (and cognitive flexibility) than do stable environments. In line with this hypothesis, the β-AR-blocker, propranolol, used to reduce anxiety and blood pressure, affects how people responded to stable situations and to changeable ones [255]. Specifically, propranolol made participants more likely to rely on their expectations, based on prior experience with two co-occurring sensations, an effect that might contribute to its anxiolytic potential. However, it also led to slower learning to adjust these expectations when contingencies were uncertain and volatile/changeable. Thus, high levels of NA, e.g., acting through β1-AR and α1-AR, may weaken established patterns of network connectivity [101], promoting new conformations needed for the updating of our beliefs based on environmental uncertainty, allowing us to be more flexible in our constantly changing world.

In summary, both DA and NA play key roles in working memory, top-down control, and cognitive flexibility. The DA and NA inverted U dose-response curves seen in animals (Figs 4–6) also apply in humans [132], with moderate levels enhancing PFC functions, while high levels of catecholamine release, e.g., during stress, impairing dlPFC functions [256]. The separation of beneficial vs. detrimental NA actions by receptor subtype (α2A-AR vs. α1-AR respectively) has speeded the successful translation of therapeutics from animals to humans, while the D1R inverted U dose-response at the same receptors will remain a challenge. While it is clear that there is considerable specificity when it comes to these different neuromodulators, the precise nature of this specificity remains to be elucidated in humans. The use of more selective receptor agents, like the novel non-catechol D1 receptor selective and D2 receptor selective agents [206], ideally in pretreatment designs [184], and direct comparison of noradrenergic vs. DAergic agents (such as sulpiride vs. propranolol [257, 258]), should help to disentangle their likely complementary roles in future studies. There are certainly hints in the literature, with, e.g., NA subserving value-free, random forms of exploration [259] vs. DA subserving more informed, directed forms of exploration [260]. However, it will be important to remember that DA and NA have differing effects through different receptors, and that many compounds available for human use are nonselective. In particular, there is a great need for selective, low affinity D1R agonists, and selective D1R antagonists, that are safe to use in humans, to truly understand DA actions on human cognitive circuits, particularly as this receptor predominates in PFC circuits.

### Effects of acetylcholine on human PFC function

DA and NA are not the only neuromodulators that modulate PFC function, such as attention shifting, behavioral flexibility and working memory. ACh is also released in the PFC upon the presentation of a salient target, and pharmacological manipulations of ACh in humans have also revealed inverted U-shaped relationship with task performance [261] (see above). Cholinergic receptor stimulation in PFC is thought to elicit an attentional shift akin to Posner’s attentional orienting response, in order to align attention with a source of sensory input. Much of the recent cognitive work on ACh in humans has focused on perceptual representations in visual cortex. For example, cholinergic enhancement with the cholinesterase inhibitor donepezil decreases the spread of early visual cortical excitatory fMRI responses to a visual stimulus [262] consistent with cholinergic receptor enrichment on interneurons in V1 [263], and improves detection of a target flanked by distractors, consistent with sharpened visuospatial perceptual representations [264], and in line with cholinergic agents reducing responses to distractors in monkey dlPFC neurons [265]. Similar effects were not seen after administration of bromocriptine (a DA D2/D1 receptor agonist) or guanfacine (a noradrenergic α2A-AR agonist) [264], suggesting a specific role of acetylcholine in sharpening perceptual representations.

In addition, cholinergic modulation also influences human working memory. For example, blockade of muscarinic and nicotinic receptors impairs working memory performance [167], while a nicotinic agonist can improve working memory in both non-smokers and abstinent smokers [266, 267]. Nonspecific enhancement of cholinergic signaling can also enhance the processing of the posterior cortices, e.g., sensory input from the thalamus to the visual cortices, but high doses can weaken top-down internal models held online by the PFC [261, 268]. In contrast to NA manipulations, cholinergic manipulations generally leave extra-dimensional set-shifting unaffected. Conversely, cholinergic changes in rodents are associated with attentional shifts in Posner-like attention orienting paradigms where subjects are aware of cue invalidity [269]. These involve parietal attentional mechanisms [270] that are impaired by cholinergic lesions in monkeys [271]. Moreover late (but not early) reversal learning is sensitive to manipulation of ACh, but not NA [66, 246]. Thus, the signals that trigger this NA- and Ach-mediated flexibility might differ. As suggested by Yu and Dayan (2005): NA might be involved predominantly when changes in the environment are “unexpected,” whereas ACh might instead signal “expected” uncertainty, which arises from known unreliability of predictive relationships within a familiar environment [272].

### Effects of serotonin on human PFC function

Functions associated with the dlPFC, such as spatial working memory and attentional set-shifting, have been the focus of only few human studies on serotonin. Early work revealed that the serotonin releaser fenfluramine impaired spatial delayed response task performance in contrast to the DA agonist bromocriptine, which facilitated spatial memory [177]. Decrements in working memory performance have been observed after increases in the serotonin precursor tryptophan [273] and theoretical work has highlighted similar nonlinear complexities as for the DA system [274]. As is the case for nonhuman primate studies, human studies have revealed that functions associated with the medial and orbital frontal cortex, such as probabilistic reversal learning tasks, are particularly sensitive to serotonergic manipulation or stratification [246, 275, 276]. Conversely, acute tryptophan depletion does not affect attentional set-shifting [277, 278]. In line with these observations, the majority of studies on serotonin’s role in human cognition and behavior have focused on affective (hardwired aversive) biases of learning and decision making, often as a function of delay, risk or effort costs [279–284]. Serotoninergic changes in aversive biases can surface as changes in inhibitory tendencies [285–287]. For example, under basal conditions, humans respond more slowly to an aversive stimulus than a rewarding stimulus, but following tryptophan depletion, they respond equally quickly to both [285]. However, unlike DA or NA, manipulating global serotonin levels does not affect performance on tasks of inhibition that have no clear affective component, such as the stop-signal reaction time task [246, 288–291], and the go/nogo task [292, 293] (but see [294]). These findings are consistent with what is seen in monkeys, where serotonin depletion alters the functioning of orbital PFC, but not dlPFC (see above). These associations reflect serotonin’s implications in disorders of impulsivity and depression, but the jury is still out regarding the direction of serotonin’s effects, or the selectivity to the aversive domain [295]. It is likely that the effects vary between subcortical and cortical target systems [296], e.g., as a function of environmental threat levels [297] and/or the degree to which stressors are controllable [298]. It is also likely that manipulations that influence selective serotonergic receptors may have a very different, and larger effect than global changes in serotonin availability, but these compounds are rarely available for human use. How these compounds alter medial PFC and cingulate functioning may have particular relevance to the etiology and treatment of mood disorders, and will be especially important for future research.

### Summary of human data

This review of the effects of manipulating the monoamine and cholinergic systems on human cognition reveals a number of general principles of chemical neuromodulation that have also been noted in computational theories of neuromodulation [299, 300]. First, manipulation of these large ascending neuromodulators has radically different functional consequences depending on where they act in the brain. Second, all these systems exhibit nonlinear Inverted U shape functions and baseline dependency: drugs that increase receptor stimulation having positive effects in systems with low baseline levels of activity, but negative effects in systems with high baseline levels of activity. While such nonlinearity is a feature of any system that is geared toward self-regulation or homeostasis (including body temperature and home central heating), it is particularly apt for the neuromodulation of the PFC, where inverted U dose-response curves are often remarkably narrow, creating a challenge for pharmacological treatments. The implication of this observation is that isolation of drug effects on human cognition requires that the baseline state of the system is taken into account. Finally, release of and responsiveness to these neuromodulators are all regulated not only by the activity of the neurons in the brainstem, but also locally in their target brain regions. This allows the impact of these neuromodulatory systems to be controlled in a top-down manner by advanced cortical computations of current beliefs about the environment. Future studies might address the obvious next question whether our remarkable capacity to adapt flexibly to our constantly changing environment stems in part from this ability to prioritize distinct neurochemical projection systems in a manner that is tailored to the current perceived environment.

### Future directions and clinical implications

Research in both human and nonhuman primates finds that the arousal neuromodulators have powerful and complex effects on PFC circuits, often with different effects based on amount of release, and with different cognitive operations having distinct neurochemical needs. Most modulators have a narrow inverted U dose-response influence on PFC functions, which coordinates arousal and cognitive states. However, these actions are often the cause of cognitive deficits, especially as stress potentiates mental disorders, and have complicated the translation of potential therapeutics from basic to clinical research. Additional pharmacological tools (receptor selective, low affinity matching endogenous transmitter actions) are needed to better understand receptor actions, and to allow broader inverted U dose-response curves, which may facilitate therapeutic strategies. There are many outstanding questions in this field, especially as research to date in nonhuman primates as only been performed in a few labs, and focused on just a few PFC subregions. Important, outstanding questions include:

- What are the roles of neuromodulators in dlPFC layers beyond layer III and on GABA interneurons?
- How do neuromodulators impact the functioning of PFC areas beyond the dlPFC? In particular, what are the roles of various 5HT receptors on neuronal firing in BA25 and on stress-related computations?
- How do the molecular cellular signaling mechanisms and the computational features of DA’s effects on working memory and the cortical hierarchy differ from those of NA and how are the various systems controlled/prioritized?
- How do the various neuromodulators implicated in working memory and goal-directed behavior interact synergistically and/or competitively?
- Can we expand our use of genetic tools in the primate to produce circuit and cell-specific manipulations, similar to the powerful studies now being done in rodent? These new technologies also allow visualization of neuromodulator release. Can we create transgenic monkeys to have a better understanding of how mutations associated with cognitive disorders alter the circuits that mediate higher cognition?
- Can we develop new methods to target drugs to specific brain regions in humans? Can we find unique signatures of PFC subcircuits that might allow nanoparticle targeting of drug deliver?
- Can we begin to understand the neuromodulation of PFC metacognitive operations such as insight, which are impaired by uncontrollable stress [301], often at great cost to society, e.g., as is evident in the COVID pandemic. Might these capabilities, generated by the frontal pole, be modulated in a similar of differing fashion to the dlPFC?

Given the power of neuromodulatory influences on the primate PFC, understanding these mechanisms will be essential to revealing the etiology of many cognitive disorders, and hopes for superior therapeutics.
